# Acute myeloid leukemia following icotinib therapy for non-small cell lung cancer: a case report and literature review

**DOI:** 10.3389/fonc.2026.1967553

**Published:** 2026-10-22

**Authors:** Huiling Huang, Lu Song, Fangqin Tong, Lei Li

**Affiliations:** 1 Department of Pharmacy, Wuhan Asia General Hospital, Wuhan, China; 2 Department of Pharmacy, Liyuan Hospital of Tongji Medical College of Huazhong University of Science & Technology, Wuhan, China; 3 Department of Oncology, Wuhan Asia General Hospital, Wuhan, China

**Keywords:** adverse drug reaction, epidermal growth factor receptor tyrosine kinase inhibitors, icotinib, non-small cell lung cancer, treatment-related myeloid neoplasm

## Abstract

Icotinib significantly improves survival and prognosis in patients with epidermal growth factor receptor (EGFR)-sensitive non-small cell lung cancer (NSCLC). It is generally well-tolerated, with treatment-related myeloid neoplasm (t-MN) being a rare adverse reaction. We report a case of a 75-year-old female patient with lung adenocarcinoma (EGFR exon 21 L858R mutation) who underwent four cycles of pemetrexed combined with platinum-based chemotherapy postoperatively, followed by radiation therapy to the supraclavicular and mediastinal lymphatic drainage regions, and sequential targeted therapy with icotinib. Approximately 32 months after initiating icotinib, the patient developed severe anemia, leading to discontinuation of the drug. She was subsequently diagnosed with acute myeloid leukemia (AML) and treated with a regimen of lisaftoclax combined with azacitidine; bone marrow examination after one cycle of treatment showed partial remission. The patient later experienced relapse and progression of AML and died 4 months after the AML diagnosis. We also reviewed previous cases of t-MN induced during treatment with epidermal growth factor receptor tyrosine kinase inhibitors (EGFR-TKIs). Based on this literature review, it remains unclear whether EGFR-TKIs are independent risk factors for t-MN. This study suggests that patients with NSCLC receiving EGFR-TKIs should undergo long-term, regular monitoring of complete blood counts. Clinicians should be vigilant for the risk of t-MN in patients with persistent cytopenia and should identify such cases and intervene as early as possible.

## 1 Introduction

Treatment-related myeloid neoplasms (t-MNs) are a distinct subtype of disease clearly defined in the 2016 World Health Organization (WHO) classification system for myeloid neoplasms. They primarily include two categories: treatment-related myelodysplastic syndrome (t-MDS) and treatment-related acute myeloid leukemia (t-AML), which are mostly severe hematologic complications that develop months to years after cancer radiotherapy or chemotherapy (1). The advent of epidermal growth factor receptor tyrosine kinase inhibitors (EGFR-TKIs) has ushered in an era of precision targeted therapy for non-small cell lung cancer (NSCLC). Compared with traditional systemic chemotherapy, these drugs can significantly improve clinical outcomes and long-term survival in patients with epidermal growth factor receptor (EGFR)-driven NSCLC, effectively reducing treatment-related adverse reactions (2). Common adverse reactions to EGFR-TKIs primarily involve cutaneous, pulmonary, and gastrointestinal toxicity; hematologic toxicity, such as cytopenia, is not a typical side effect of this class of drugs (3). Currently, clinical reports of t-MN developing during EGFR-TKI therapy are relatively rare. This article reports a rare case of a patient with NSCLC who developed t-AML after 32 months of long-term targeted therapy with icotinib following radiotherapy and chemotherapy. Furthermore, we review the relevant literature on t-MN that occurs during EGFR-TKI treatment, aiming to provide a reference for the safe clinical use of EGFR-TKIs and for the prevention and control of long-term complications.

## 2 Case presentation

A 75-year-old female patient underwent thoracoscopic left upper lobectomy with hilar and mediastinal lymph node dissection on January 10, 2023, following a chest computed tomography (CT) scan that revealed a pulmonary mass. Postoperative pathology revealed infiltrative adenocarcinoma of the lung (moderately differentiated, with 45% acinar, 45% papillary, and 10% lepidic patterns); metastases were found in 2, 1, and 1 lymph nodes in groups 5, 6, and 10, respectively. Genetic testing revealed an L858R mutation in exon 21 of the EGFR gene. A follow-up examination in February 2023 suggested a possible secondary malignant tumor in the left frontal lobe, upstaged to clinical stage IV. On February 14, 2023, the patient began three cycles of chemotherapy with pemetrexed combined with cisplatin. Before the initiation of chemotherapy, the white blood cell count was 6.91 × 10^9^/L, the hemoglobin level was 121 g/L, the platelet count was 203 × 10^9^/L, the serum creatinine was 67 μmol/L, and the estimated glomerular filtration rate (eGFR) was 79 mL/min. On April 19, 2023, during the fourth cycle of chemotherapy, cisplatin was replaced with nedaplatin because of renal impairment that had developed after three cycles of cisplatin-based chemotherapy (serum creatinine, 107 μmol/L; eGFR, 45 mL/min). On May 12, 2023, the patient began radiation therapy (50 Gy/25 fractions of 2 Gy each) to the supraclavicular and mediastinal lymph node drainage areas. Before the initiation of icotinib on May 22, 2023, the white blood cell count was 6.42 × 10^9^/L, the hemoglobin level was 102 g/L, and the platelet count was 376 × 10^9^/L. The patient then began targeted therapy with oral icotinib (125 mg three times daily).

On November 3, 2025, the patient was admitted to the hospital for chest tightness, shortness of breath, and fatigue. A complete blood count revealed a hemoglobin (Hb) level of 41 g/L, a white blood cell (WBC) count of 3.11 × 10^9^/L, and a platelet (PLT) count of 190 × 10^9^/L. Clinical symptoms improved following symptomatic treatment. The antitumor treatment was assessed as achieving disease stabilization, and the patient continued oral icotinib therapy. On December 8, 2025, the patient was readmitted for symptom recurrence. A complete blood count showed hemoglobin 39 g/L, white blood cell count 11.27 × 10^9^/L, and platelet count 162 × 10^9^/L. Following a blood transfusion, the hemoglobin level rose to 81 g/L. Painless gastrointestinal endoscopy ruled out gastrointestinal tumors and chronic gastrointestinal bleeding. Given the patient’s long history of targeted therapy, a drug-related hematologic disorder was initially suspected, and icotinib was discontinued immediately.

On January 6, 2026, the patient presented to the Department of Hematology. A complete blood count showed hemoglobin at 74 g/L, a white blood cell count of 85.6 × 10^9^/L, and a platelet count of 62 × 10^9^/L. Bone marrow aspirate morphology revealed: markedly active proliferation of nucleated cells in the bone marrow; the granulocyte lineage accounted for 60%, with most granules enlarged and coarsened; blast cells accounted for 21%, with cells of varying sizes, scant cytoplasm stained pale blue to blue, and a few cells showing small azurophilic granules in the cytoplasm; nuclei were round or irregular in shape; nuclear chromatin was uniform and fine, with nucleoli of varying prominence; monocytes accounted for 14%, and monoblasts accounted for 8%. The blood smear showed mature red blood cells of varying sizes, with some oval-shaped cells; platelets were scattered and rare, with occasional large platelets. Immunohistochemical staining showed POX 36% (+) and ANAE 25% (+). Bone marrow biopsy revealed: extremely active proliferation of hematopoietic tissue, with nucleated cells occupying >95% of the trabecular spaces; proliferation of immature cells, distributed sporadically or in clusters; megakaryocytes were visible, distributed sporadically, with occasional single-nucleated and double-nucleated megakaryocytes; occasional small clusters of lymphocytes; a relatively high proportion of monocytes; trabeculae were normal. Immunophenotyping by bone marrow flow cytometry revealed that nucleated cells were phenotypically abnormal myeloid progenitor cells expressing CD34, CD117, CD13, CD38 (partial expression), and HLA-DR, with a small proportion expressing CD33; the granulocyte lineage showed abnormal development; the proportion of monocytes was elevated, predominantly consisting of mature cells; no obvious abnormalities were observed in the lymphocyte phenotype. Genetic mutation screening revealed mutations in *PPM1D* (exon 6 c.1714C>T, VAF 48.8%), *TET2* compound heterozygous mutations [exon 8 c.4021G>C (p.A1341P, VAF 46.8%) and exon 3 c.1337del (p.L446*7, VAF 44.4%)], *DNMT3A* (exon 10 c.1238dup, VAF 44.6%), and *RUNX1* (exon 8 c.941_942insG, VAF 38.3%). Chromosomal karyotype analysis showed 45,XX,−7[20]. Screening for leukemia fusion genes was negative for all tested fusion genes.

The patient received a standard cytotoxic chemotherapy regimen for non-small cell lung cancer, but completed only 4 cycles of treatment. No severe myelosuppression occurred following any of the chemotherapy cycles or the course of radiotherapy, and the patient had no family history of hematologic malignancies. According to the 2016 WHO classification, AML is defined by the presence of ≥20% blasts in the bone marrow or peripheral blood (1). In this patient, bone marrow blasts accounted for 21%, thus fulfilling the diagnostic threshold for AML. A definitive diagnosis of acute myeloid leukemia (AML) was made, with treatment-related acute myeloid leukemia considered. However, given the patient’s history of multiple exposures to cytotoxic chemotherapy, radiation therapy, and long-term EGFR-TKI targeted therapy, further evidence is needed to confirm whether this myeloid lesion was induced solely by EGFR-TKI. The causal relationship between icotinib and the t-MN in this case was assessed using the Naranjo Assessment Scale, yielding a total score of 4 points; the association was judged to be “possible” (**Table 1**).

**Table 1:** Naranjo score for the association between icotinib and the treatment-related myeloid neoplasm.

Item	Yes	No	Do not know/not done	Score	Rationale
1. Are there previous conclusive reports on this reaction?	+1	0	0	+1 (Yes)	Eight published reports (13 cases) of myeloid neoplasms arising during or after EGFR-TKI therapy were identified in the literature review presented here, including one case that arose during icotinib therapy. Well-documented published case reports of this reaction are therefore available in the literature.
2. Did the adverse event appear after the suspected drug was administered?	+2	−1	0	+2 (Yes)	AML was diagnosed during continuous icotinib treatment (month 32); the temporal-sequence criterion is met.
3. Did the adverse reaction improve when the drug was discontinued or a specific antagonist was administered?	+1	0	0	0 (Do not know)	This item is not attributable: the adverse event under assessment is a myeloid neoplasm, a clonal disease with a latency of months to years; even if the neoplasm had been promoted by icotinib, discontinuation of the drug alone could not have improved it, and anti-leukemic therapy is required. The withdrawal–improvement (dechallenge) structure on which this item rests is therefore not applicable to this disease, in contrast to ordinary adverse drug reactions. The clinical course was consistent with this: during the post-withdrawal interval only supportive measures were given and the myeloid neoplasm was progressing; this should not be read as a negative dechallenge. No specific antagonist of icotinib exists.
4. Did the adverse reaction reappear when the drug was re-administered?	+2	−1	0	0 (not done)	Icotinib was not re-administered; not assessable.
5. Are there alternative causes (other than the drug) that could on their own have caused the reaction?	−1	+2	0	0 (Do not know)	Although prior chemotherapy and radiotherapy are plausible relevant exposures in this patient, neither was followed by significant myelosuppression, and the myeloid neoplasm emerged while icotinib was being given continuously. Whether those exposures could, on their own, have caused this reaction cannot be determined retrospectively, and a *de novo* origin cannot be excluded either, because the independent sufficiency of an alternative cause could not be established.
6. Did the reaction reappear when a placebo was given?	−1	+1	0	0 (not done)	Placebo was not administered.
7. Was the drug detected in the blood (or other fluids) in concentrations known to be toxic?	+1	0	0	0 (not done)	Therapeutic drug monitoring was not performed.
8. Was the reaction more severe when the dose was increased, or less severe when the dose was decreased?	+1	0	0	0 (not done)	Icotinib was given at a constant dose throughout; no dose adjustment was made.
9. Did the patient have a similar reaction to the same or similar drugs in any previous exposure?	+1	0	0	0 (No)	Icotinib was the first and only EGFR-TKI the patient had ever received, and there was no documented history of a similar reaction to related agents.
10. Was the adverse event confirmed by any objective evidence?	+1	0	0	+1 (Yes)	The diagnosis of acute myeloid leukemia was confirmed by bone marrow morphology and immunophenotyping.
Total				4 (possible)	

Scores in the “Yes/No/Do not know or not done” columns are the possible scores for each item; the answer given in this case is shown in parentheses in the “Score” column. Interpretation: ≥9 definite, 5–8 probable, 1–4 possible, ≤0 doubtful.

Following diagnosis, the patient was treated with a regimen of lisaftoclax combined with azacitidine. After one cycle of treatment, the patient developed severe bone marrow suppression (hemoglobin 42 g/L, platelet count 12 × 10^9^/L); a follow-up bone marrow examination showed partial remission of AML. Subsequently, due to AML relapse and progression, the patient died 4 months after the diagnosis of AML. Serial complete blood count parameters recorded from November 3, 2025, to January 30, 2026 are summarized in **Figure 1**.

**Figure 1:**
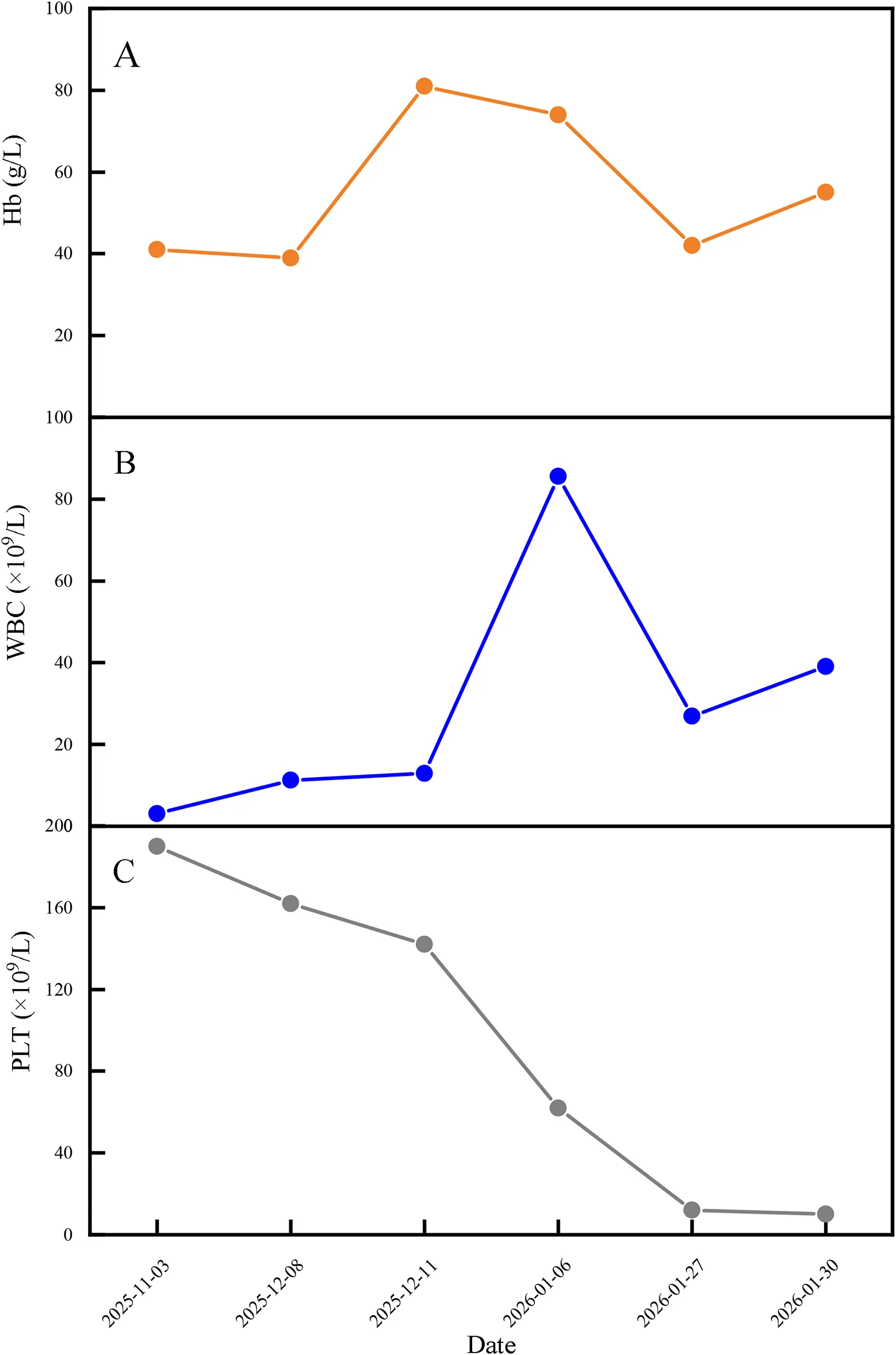
Dynamic changes in complete blood count parameters from the onset of cytopenia to after the first cycle of treatment. **(A)** Hemoglobin (Hb) levels; **(B)** White blood cell (WBC) counts; **(C)** Platelet (PLT) counts.

## 3 Discussion

t-MN is a rare and life-threatening complication following anticancer therapy, with an overall incidence of 0.13 cases per 100,000 population, as well as significant variation by age, race, and calendar period (4). t-MN most commonly develops following cytotoxic chemotherapy and/or radiation therapy; various cytotoxic agents, including alkylating agents, topoisomerase II inhibitors, antimetabolites, and anti-microtubule agents, have been associated with the onset of t-MN (5). In recent years, poly(ADP-ribose) polymerase (PARP) inhibitors and chimeric antigen receptor T-cell (CAR-T) therapy have also been reported to induce t-MN (6–9).

With the widespread use of EGFR-TKIs in the treatment of NSCLC, EGFR-TKI-associated t-MN has gradually garnered clinical attention. A comprehensive literature search was conducted in PubMed, Web of Science (Core Collection), Embase, the China National Knowledge Infrastructure (CNKI), and the Wanfang Database, covering the period from each database’s inception to June 30, 2026. The following terms were used in combination: (“EGFR-TKI” OR “epidermal growth factor receptor tyrosine kinase inhibitor” OR gefitinib OR erlotinib OR osimertinib OR icotinib OR afatinib OR dacomitinib) AND (leukemia OR “acute myeloid leukemia” OR “myelodysplastic syndrome” OR “myeloid neoplasm” OR “treatment-related myeloid neoplasm” OR “hematologic neoplasia” OR “hematologic malignancy” OR “chronic myelomonocytic leukemia” OR “myeloproliferative neoplasm”). Reports published in English or Chinese were considered. The search identified 1,230 records; after removal of 381 duplicates, 849 records were screened by title and abstract, of which 829 were excluded, and 20 full-text reports were assessed for eligibility. Cases were included if they fulfilled all of the following criteria: (i) histologically or cytologically confirmed NSCLC; (ii) receipt of an EGFR-TKI prior to or at the time of the hematological event; (iii) a diagnosis of a myeloid neoplasm established according to the WHO classification of hematopoietic and lymphoid tumors (including MDS, AML, CML, or other myeloid neoplasms) that arose during or after EGFR-TKI exposure, with or without prior cytotoxic chemotherapy and/or radiotherapy, and for which (a) the original authors themselves favored a therapy-related attribution — that is, they designated the neoplasm as therapy-related, treatment-associated, or treatment-emergent rather than as a *de novo* or independent second primary malignancy — and (b) the EGFR-TKI was put forward by those authors as a possible contributing or risk factor, or its possible role was expressly left in doubt; and (iv) availability of individual-level clinical details (EGFR-TKI exposure and latency, disease type, karyotype and/or molecular findings, treatment, and outcome where available). Reviews, editorials, conference abstracts, and duplicate publications were excluded, and for duplicated cases only the most complete report was retained. All candidate reports were independently screened by two authors (HH and LS); any discrepancy was resolved by discussion, and where this did not lead to agreement, by adjudication of the senior author (LL). Ultimately, 8 articles (1 in Chinese and 7 in English) reporting 13 cases met the criteria and were included (10–17).

A literature analysis was conducted on a total of 14 cases (including the present case; see **Table 2** for details): (1) Population and Exposure Factors: The primary tumors were predominantly EGFR L858R-mutated lung adenocarcinomas; most patients had previously received multiple cycles of platinum-based chemotherapy and localized radiotherapy to the chest, bones, or brain; the EGFR-TKIs involved included gefitinib (5 cases), erlotinib (5 cases), osimertinib (2 cases), and icotinib (2 cases); the duration of treatment ranged from 4 to 32 months, with a median latency period of approximately 10 months. Latency was defined as the interval from the initiation of EGFR-TKI therapy to the diagnosis of t-MN, and this definition was applied consistently to all reviewed cases. (2) Disease Type: t-AML was the most common, with a total of 8 cases; among these, treatment-associated acute promyelocytic leukemia (t-APL) accounted for more than half of the t-AML cases and was a characteristic subtype; there were 4 cases of t-MDS and 2 cases of treatment-associated chronic myeloid leukemia (t-CML). (3) Immunophenotype: Both cases of icotinib-associated myeloid neoplasms (1 case of t-MDS and 1 case of t-AML) co-expressed the myeloid antigens CD34, CD117, HLA-DR, CD13, and CD33; ectopic expression of the mature granulocyte antigen CD16 was observed in t-MDS; t-APL specifically expressed only CD13 and CD33, with no abnormal co-expression of CD16. (4) Chromosome Karyotype: EGFR-TKI-associated t-MN cases exhibited diverse karyotypes, ranging from characteristic balanced translocations such as t(9;22), t(15;17), and inv(16) to numerical abnormalities (+8, +21), chromosomal deletions (7q, 20q), isolated monosomy 7, and normal karyotypes. (5) Gene mutations: In two cases of icotinib-associated t-MN, myeloid-related mutations in *DNMT3A*, *TET2*, *EZH2*, *RUNX1*, and *PPM1D* were detected. (6) Complete blood count: Most patients presented with monolineage or multilineage cytopenia, with anemia and severe thrombocytopenia being the most common. (7) Treatment and Prognosis: Clinically, the preferred approach is to discontinue EGFR-TKIs and initiate specialized treatment for myeloid neoplasms as early as possible; for t-APL, regimens primarily based on all-trans retinoic acid (ATRA) are used, with a generally favorable prognosis; for t-MDS, demethylating agents such as azacitidine are commonly selected; patients receiving only supportive care have a poor prognosis; t-AML cases with an isolated monosomy 7 and multiple myeloid mutations are highly malignant, progress rapidly, and have significantly shortened survival. Most cases in this cohort had a history of radiotherapy and chemotherapy, making it impossible to fully distinguish the causative factors; however, the presence of two t-MN cases treated with EGFR-TKIs alone suggests that EGFR-TKIs may pose a risk of inducing t-MN, a possibility that should not be overlooked clinically.

**Table 2:** Literature review of t-MN in NSCLC patients following EGFR-TKI treatment .

Author/Year	Age/Sex	Pathological type	EGFR genotype	Prior chemotherapy	Prior radiotherapy	EGFR-TKI agent	Latency from TKI initiation	t-MN subtype	Key characteristics of t-MN (immunophenotype/cytogenetics/mutations)	WBC (10^9^/L), Hb (g/L), and PLT (10^9^/L) at t-MN diagnosis	Treatment for t-MN	Whether EGFR-TKI was continued	Outcome
Leng et al. (2024) (10)^†‡^	82/F	Adenocarcinoma	L858R	No	No	Icotinib, 4 mo	4 mo	MDS	CD34 (+), CD13 (+), CD33 (+), CD117 (+), HLA-DR (+). Immunophenotype: CD16, CD13. Chromosome karyotype: 47,XX,+8[20]. Gene mutations: *DNMT3A*, *EZH2*	WBC 2.5, Hb 87, PLT 225	Azacitidine	Yes	Complete remission of MDS; no progression of lung cancer
Zhang et al. (2024) (11)^†‡^	45/M	Adenocarcinoma	L858R	No	No	Osimertinib, 8 mo	8 mo	CML	Chromosome karyotype: 46,XY,t(9;22)(q34.1;q11)[3]. BCR-ABL(210) (+)	WBC 28.31, PLT 733	Flumatinib	Yes	CML and lung cancer remained stable
Koo et al. (2021) (12)	52/M	Adenocarcinoma	L858R	6 cycles of PTX and CBP, 3 cycles of GEM and NVB	Multiple brain metastatic lesions (total 23 Gy); whole-brain (total 30 Gy)	Erlotinib, 11 mo	11 mo	AML	Chromosome karyotype: 46,XY,inv(1)(p22q32), inv(16)(p13.1q22). CBFB/MYH11 rearrangement	WBC 7.6, Hb 124, PLT 83	Daunorubicin/Cytarabine; Mitoxantrone/Etoposide	No	Recurrence of AML; progression of lung cancer; died of respiratory failure 10 months after receiving the AML diagnosis
Tian et al. (2023) (13)^†^	58/F	Adenocarcinoma	L858R	No	Thoracic vertebrae T5 (3,000 cGy 10 times, 2,100 cGy 7 times); T11 (3,000 cGy 10 times, 2,100 cGy 7 times)	Osimertinib, 13 mo	13 mo	AML (APL)	Chromosome karyotype: 46,XX,add(7,11), t(15;17)(q24;q21)[6]/46,XX[14]. PML/RARα (Bcr3) (+)	WBC 1.49, Hb 96, PLT 97	All-trans retinoic acid/Arsenic acid	No	Following remission of APL, the patient received chemotherapy for lung cancer
Stathopoulos et al. (2010) (14)^‡^	67/M	Adenocarcinoma	NA	6 cycles of GEM and DDP	No	Erlotinib, 4 mo	8 mo	MDS	Chromosome karyotype: 46,XY,del(20)(q11)[15]/47,idem,+21[7]/48,idem,21,+21[-3]	Grade 4 thrombocytopenia	NA	No	NA
Stathopoulos et al. (2010) (14)^‡^	70/M	Adenocarcinoma	NA	6 cycles of PTX and DDP	No	Erlotinib, 8 mo	8 mo	CML	BCR-ABL (+)	WBC 92	NA	No	NA
Stathopoulos et al. (2010) (14)	60/F	Adenocarcinoma	NA	6 cycles of NVB and DDP; 3 cycles of VP-16 and CBP	Primary lung lesions and mediastinum	Erlotinib, 8.5 mo	8.5 mo	AML	Chromosome karyotype: 46,XX,del(7)(q22),add(21)(q22)	WBC 2.5, PLT 72	Best supportive care	NA	Died of pneumonia 3.5 months after diagnosis of AML
Stathopoulos et al. (2010) (14)^‡^	59/F	Squamous cell carcinoma	NA	6 cycles of VP-16 and CBP	No	Erlotinib, 5.5 mo	5.5 mo	MDS	Chromosome karyotype: 47,XX,+8, t(5;9)(q13;q34)	WBC 3.2	Best supportive care	Yes	Died of respiratory failure due to lung cancer progression, 8 months later
Ravindranathan et al. (2007) (15)	72/M	Adenocarcinoma	NA	5 cycles of GEM and CBP	Area of spinal cord compression (total 30 Gy)	Gefitinib, 6 mo	6 mo	MDS	Chromosome karyotype: normal karyotype (46, XY)	Trilineage myelosuppression	Azacitidine	No	Died due to the progression of lung cancer
Ennishi et al. (2006) (16)	51/F	Adenocarcinoma	NA	6 cycles of PTX and CBP	Mediastinal lymph node (total 60 Gy)	Gefitinib, 14 mo	14 mo	AML (APL)	CD13 (+), CD33 (+). Chromosome karyotype: t(15;17)(q22;q21). PML/RARα (+)	WBC 2.3, Hb 132, PLT 15.2	All-trans retinoic acid/Idarubicin/Cytarabine	No	Lung cancer progression
Matsuo et al. (2006) (17)	49/F	Adenocarcinoma	NA	2 cycles of DOC, DDP, and CPT-11	Brain metastases (total 22.933 Gy)	Gefitinib, 15 mo	15 mo	AML (APL)	CD13 (+), CD33 (+). Chromosome karyotype: normal karyotype (46, XX). PML/RARα (+)	WBC 3.4, Hb 107, PLT 13.5	All-trans retinoic acid/Cytarabine/Mitoxantrone	Yes	Lung cancer remained stable
Matsuo et al. (2006) (17)	65/M	Squamous cell carcinoma	NA	2 cycles of DDP, MMC, and NVB; Uracil/tegafur 2 mo	Fractionated radiotherapy (2 Gy × 25)	Gefitinib, 25 mo	25 mo	AML (APL)	CD13 (+), CD33 (+). Chromosome karyotype: normal karyotype (46, XY). PML/RARα (+)	WBC 1.9, Hb 124, PLT 14.4	All-trans retinoic acid/Anthracyclines/Cytarabine	NA	Lung cancer remained stable
Matsuo et al. (2006) (17)^‡^	72/M	Adenocarcinoma	NA	PTX and CBP; 10 cycles of chemotherapy: DDP, CPT-11, DOC, GEM, and NVB; 3 cycles of CPT-11 and ambruticin	No	Gefitinib, 9 mo	26 mo	AML (APL)	CD13 (+), CD33 (+). Chromosome karyotype: normal karyotype (46, XY). PML/RARα (+)	WBC 1.9, Hb 129, PLT 13.3	All-trans retinoic acid/Cytarabine	NA	Progression of lung cancer; died due to APL recurrence
Present case	75/F	Adenocarcinoma	L858R	4 cycles of PEM and DDP/NDP	Supraclavicular and mediastinal lymphatic drainage regions (2 Gy × 25)	Icotinib, 32 mo	32 mo	AML	CD34 (+), CD117 (+), CD13 (+), CD38 (partial +), HLA-DR (+), CD33 (+). Chromosome karyotype: 45,XX,-7[20]. Gene mutations: *PPM1D, TET2, DNMT3A, RUNX1*	WBC 85.6, Hb 74, PLT 62	Lisaftoclax/Azacitidine	No	Recurrence and progression of AML; died 4 months after AML diagnosis

EGFR, Epidermal growth factor receptor; EGFR-TKI, Epidermal growth factor receptor tyrosine kinase inhibitor; NSCLC, Non-small cell lung cancer; t-MN, Treatment-related myeloid neoplasm; MDS, Myelodysplastic syndrome; CML, Chronic myeloid leukemia; AML, Acute myeloid leukemia; APL, Acute promyelocytic leukemia; WBC, White blood cell; Hb, Hemoglobin; PLT, Platelet; NA, Not Available. PTX, paclitaxel; CBP, carboplatin; GEM, gemcitabine; NVB, vinorelbine; DDP, cisplatin; VP-16, etoposide; DOC, docetaxel; CPT-11, irinotecan; MMC, Mitomycin C; PEM, pemetrexed; NDP, nedaplatin.

^†^ No prior chemotherapy before the EGFR-TKI. ^‡^No prior radiotherapy before the EGFR-TKI. ^†‡^Neither prior chemotherapy nor prior radiotherapy; the EGFR-TKI was the only cytotoxic exposure before the myeloid neoplasm developed.

Regarding the latency period, a study by Radivoyevitch et al. indicated that in patients with non-hematologic malignancies who underwent radiotherapy with or without chemotherapy, the risk of acute myeloid leukemia (AML) and myelodysplastic syndrome (MDS) begins to rise 9–12 months after treatment, peaks at 1.5–2.5 years, and returns to the level of the general population after 10–15 years (18). In patients treated with topoisomerase II inhibitors, the latency period for t-MN is typically shorter (1–2 years), whereas patients treated with alkylating agents and/or radiation therapy have a relatively longer latency period (5 years on average) (19). The median latency period for PARP inhibitor-associated t-MN is 17.8 months (6). The median latency period for CAR-T cell therapy-associated t-MN is 6–10 months (7–9). Based on a comprehensive review of the existing literature, the median latency period for EGFR-TKI-induced t-MN is 10 months, which is shorter than that for t-MN induced by conventional chemotherapy drugs and radiation therapy.

The pathogenesis of t-MN is extremely complex and may involve a multistep malignant transformation process resulting from the combined effects of multiple factors, including cytotoxic therapies, genetic predisposition, clonal hematopoiesis (CH), and the bone marrow microenvironment (20). The World Health Organization’s 5th Edition Classification of Hematolymphoid Tumors defined clonal hematopoiesis for the first time in the tumor classification system as a precursor state to myeloid neoplasms; it proposed that mutated pluripotent hematopoietic stem and progenitor cells form clonal cell populations, which continuously expand due to selective proliferative advantages, thereby driving the development of myeloid neoplasms (21). However, the mechanism by which EGFR-TKIs induce treatment-related myeloid neoplasms remains unclear, and it is not yet established whether these drugs participate in the development of CH through relevant signaling pathways. Existing studies have shown that neutrophils can express the epidermal growth factor receptor, and that epidermal growth factor can enhance tumor necrosis factor-α (TNF-α)-induced neutrophil phagocytic activity; this effect can be completely blocked by epidermal growth factor receptor tyrosine kinase inhibitors (22). Other studies have confirmed that exogenous heparin-binding epidermal growth factor-like growth factor (HB-EGF) can promote the *in vitro* proliferation of bone marrow mesenchymal stem cells (MSCs) by binding to epidermal growth factor receptor 1 (HER1) on MSCs (23). Therefore, whether EGFR-TKIs contribute to the onset and progression of hematologic malignancies by inhibiting neutrophil function, blocking MSC proliferation, and disrupting the body’s immune system and bone marrow microenvironment remains to be further verified by subsequent studies. Even so, these mechanistic considerations remain speculative: a direct causal link between EGFR-TKIs and t-MN has not been proven, and the existing evidence cannot exclude confounding by prior chemotherapy and/or radiotherapy.

The overall genetic profile of t-MN is highly complex, with no single signature genetic abnormality; however, two types of characteristic chromosomal abnormalities highly associated with prior therapeutic exposure have been identified: t-MN induced by alkylating agents or radiation therapy typically presents with complete or partial long-arm deletions of chromosomes 5 and 7 (–5/del(5q), -7/del(7q)), and usually presents initially as MDS, with a tendency to rapidly progress to AML; topoisomerase II inhibitor-associated t-MN most commonly involves a *KMT2A* rearrangement at the 11q23.3 locus or a balanced translocation of *RUNX1* at the 21q22.1 locus, with most patients presenting directly with AML without a preceding MDS phase (19). Patients with t-MN commonly carry somatic mutations; studies show that the somatic mutation positivity rate is 93% for both t-MN and primary MDS, but the mutation patterns between the two are markedly different; *TP53* mutations account for 29.5% of cases in t-MN, significantly higher than the 7% observed in primary MDS, and a multivariate regression model has confirmed that *TP53* mutations are an independent poor prognostic factor for t-MN (24). *PPM1D* is another highly frequent somatic mutation in t-MN; it is detectable in approximately 20% of patients with treatment-related MDS/AML overall and is closely associated with platinum exposure (25).

The prognosis for t-MN is extremely poor, with a median overall survival of only 8–14 months; its survival rate is significantly lower than that of primary myeloid neoplasms and declines markedly with increasing age (4,26). Allogeneic hematopoietic cell transplantation (allo-HCT) is currently the only treatment with the potential to cure t-MN; the 5-year overall survival (OS) rate after transplantation is 22% (27). Hypomethylating agents (HMAs), such as azacitidine and decitabine, are important first-line treatments for myeloid neoplasms. Selective BCL-2 inhibitors can block BCL-2-mediated anti-apoptotic pathways and induce apoptosis in tumor cells. Although the first-line regimen of venetoclax combined with HMA for t-MN can improve patient survival, its overall efficacy remains limited: the combined complete remission rate is only 39.1%, and the median OS is just 7 months (28). Lisaftoclax (APG-2575) is a new-generation, highly selective BCL-2 inhibitor independently developed in China. It exhibits a stronger affinity for the BCL-2 protein, a faster onset of action, and a lower incidence of hematologic toxicities such as thrombocytopenia and neutropenia (29). Current studies show that this drug offers the advantage of rapid onset of action in patients with acute myeloid leukemia and myelodysplastic syndrome with increased blasts (MDS-IB), with remission rates ranging from 55.6% to 62.5% after one treatment cycle, and a manageable safety profile (30).

In this case, a therapy-related myeloid neoplasm (t-MN) was strongly suspected, although *de novo* AML in an elderly patient could not be completely excluded. The pooled analysis by Mauritzson et al., based on 4,230 adult AML cases, likewise showed that the cytogenetic features of t-AML differ significantly from those of *de novo* AML: unbalanced changes in general, t(1;3), der(1;7), 3p−, −5, 5q−, −7, 7q−, t(9;11), t(11;19), t(11q23), der(12p), −17, der(17p), −18, and −21 were significantly more frequent in t-AML, whereas +8 as a sole anomaly, balanced changes in general, t(8;21), t(9;22), t(15;17), inv(16), and t(21q22) were more common in *de novo* AML (31). Several features of this case support a therapy-related origin. First, loss of chromosome 7 (−7) is the typical cytogenetic change of myeloid neoplasms arising after alkylating agents or radiotherapy (19); in the pooled cohort of Mauritzson et al., −7 itself was significantly enriched in therapy-related cases (9.3% [340/3,649] of *de novo* AML vs 29% [167/581] of t-AML; P<0.001), and monosomy 7 as the sole cytogenetic abnormality was also significantly more common in t-AML (3.1%, 114/3,649 vs 8.8%, 51/581; P<0.001) (31). The isolated −7 in this patient is consistent with this enrichment. Second, a *PPM1D* mutation was detected, at a variant allele frequency as high as 48.8%. *PPM1D* mutations are significantly enriched in therapy-related myeloid neoplasms (19.5% of t-AML and 20.2% of t-MDS), whereas only 1 of 228 patients (0.4%) in a matched *de novo* AML/MDS cohort carried such a mutation, and they are significantly associated with prior platinum exposure (odds ratio 2.9; 95% CI 1.2–7.1; P = 0.004) (25); this is consistent with the four cycles of platinum-based chemotherapy that this patient received before the leukemia was diagnosed. Third, the latency and the exposure background are not commensurate: the myeloid neoplasm was diagnosed approximately 35 months after cytotoxic therapy began and approximately 32 months after icotinib was started, shorter than the average latency of approximately 5 years for alkylating agent- and radiotherapy-associated t-MN (19), whereas previous studies suggest that the latency of EGFR-TKI-associated t-MN is itself short — in the cases reviewed in this report (14 cases including the present one) the median latency was approximately 10 months, markedly shorter than that of conventional chemoradiotherapy. It should be acknowledged that the 32-month interval in this patient is longer than this median value, but it is still shorter than the approximately 5-year average that would be expected for the cytotoxic exposure; moreover, this patient received only four cycles of chemotherapy and 50 Gy of radiotherapy, completed over approximately four months, with no severe myelosuppression at any point, whereas icotinib was given continuously for as long as 32 months. It should also be pointed out that the following features are also seen in *de novo* AML in elderly patients. First, −7 alone is insufficient to establish a therapy-related origin: in *de novo* AML this abnormality represents a difference in proportion rather than in presence (3.1% [114/3,649] vs 8.8% [51/581]; P<0.001) (31); moreover, in the current WHO classification −7 is one of the defining cytogenetic abnormalities that identify “AML, myelodysplasia-related”, irrespective of previous therapy (21). Second, the patient also carries *TET2* and *DNMT3A* mutations, which are not specific to therapy-related myeloid neoplasms: in a cohort in which therapy-related and primary cases were sequenced in parallel, the mutation frequencies of *TET2*, *ASXL1*, *DNMT3A*, *RUNX1*, and *EZH2* did not differ between primary MDS and therapy-related myeloid neoplasms (24). Third, the patient is elderly (75 years), and age itself is not discriminatory: in a population-based registry of 3,363 adults with AML, the age at diagnosis did not differ between *de novo* and therapy-related AML (median age 70 years in both); t-AML accounted for about 5% of AML cases below 40 years of age and increased to 10% at 40 years and above, so that *de novo* AML remains predominant in this age group (26).

Taken together, a therapy-related myeloid neoplasm remains the leading consideration in this case, and EGFR-TKI therapy may have induced its occurrence or accelerated its progression. Because no cytogenetic or molecular feature has yet been established as characteristic of myeloid neoplasms arising after EGFR-TKI exposure, the attribution of this case to the therapy-related category is a qualitative designation based on the totality of the evidence rather than on any single abnormality, and a *de novo* origin still cannot be completely excluded; more cases and mechanistic studies are therefore still required. Unfortunately, the patient’s perspective could not be obtained for this report because the patient passed away; we acknowledge this as a limitation of the present case report.

## 4 Conclusion

This case report and literature review suggest that although EGFR-TKIs play a significant role in the treatment of NSCLC patients and generally have good safety profiles, their potential long-term hematologic toxicity should not be overlooked. Although there is currently no conclusive evidence that EGFR-TKIs are independent risk factors for t-MN, the clinical course of t-AML that developed in this patient following long-term treatment with icotinib serves as a warning that such targeted therapies may be associated with t-MN. Patients receiving EGFR-TKIs should undergo long-term, regular monitoring of complete blood counts. Clinicians must remain highly vigilant for persistent or progressive cytopenia, promptly perform bone marrow evaluations, and ensure early diagnosis and intervention to improve patient prognosis.

## Data Availability

The original contributions presented in the study are included in the article. Further inquiries can be directed to the corresponding author.
